# The Relevance of External Quality Assessment for Molecular Testing for *ALK* Positive Non-Small Cell Lung Cancer: Results from Two Pilot Rounds Show Room for Optimization

**DOI:** 10.1371/journal.pone.0112159

**Published:** 2014-11-11

**Authors:** Lien Tembuyser, Véronique Tack, Karen Zwaenepoel, Patrick Pauwels, Keith Miller, Lukas Bubendorf, Keith Kerr, Ed Schuuring, Erik Thunnissen, Elisabeth M. C. Dequeker

**Affiliations:** 1 Department of Public Health and Primary Care, Biomedical Quality Assurance Research Unit, KU Leuven – University of Leuven, Leuven, Belgium; 2 Department of Pathology, Antwerp University Hospital, Edegem, Belgium; 3 UK NEQAS ICC & ISH, London, United Kingdom; 4 Institute for Pathology, Basel University Hospital, Basel, Switzerland; 5 Department of Pathology, Aberdeen Royal Infirmary, Aberdeen, United Kingdom; 6 Department of Pathology and Medical Biology, University of Groningen, Groningen, the Netherlands; 7 Department of Pathology, VU University Medical Center, Amsterdam, The Netherlands; University of Algarve, Portugal

## Abstract

**Background and Purpose:**

Molecular profiling should be performed on all advanced non-small cell lung cancer with non-squamous histology to allow treatment selection. Currently, this should include *EGFR* mutation testing and testing for *ALK* rearrangements. *ROS1* is another emerging target. *ALK* rearrangement status is a critical biomarker to predict response to tyrosine kinase inhibitors such as crizotinib. To promote high quality testing in non-small cell lung cancer, the European Society of Pathology has introduced an external quality assessment scheme. This article summarizes the results of the first two pilot rounds organized in 2012–2013.

**Materials and Methods:**

Tissue microarray slides consisting of cell-lines and resection specimens were distributed with the request for routine *ALK* testing using IHC or FISH. Participation in *ALK* FISH testing included the interpretation of four digital FISH images.

**Results:**

Data from 173 different laboratories was obtained. Results demonstrate decreased error rates in the second round for both *ALK* FISH and *ALK* IHC, although the error rates were still high and the need for external quality assessment in laboratories performing *ALK* testing is evident. Error rates obtained by FISH were lower than by IHC. The lowest error rates were observed for the interpretation of digital FISH images.

**Conclusion:**

There was a large variety in FISH enumeration practices. Based on the results from this study, recommendations for the methodology, analysis, interpretation and result reporting were issued. External quality assessment is a crucial element to improve the quality of molecular testing.

## Introduction

Lung cancer is amongst the leading causes of cancer related mortality worldwide [1]. Approximately 85% of lung cancers are non-small cell lung cancers (NSCLC), traditionally divided into three major cell types: adenocarcinoma, squamous cell carcinoma and large cell carcinoma [2]. Over the past decade, the availability of molecular targeted therapies has increased the progression-free survival for patients with NSCLC, adenocarcinoma in particular [3]–[6].

The approach of using biomarkers to select treatments that are tailored to individual patient profiles is referred to as precision medicine. In advanced NSCLC, *EGFR* gene mutations and *ALK* rearrangements are currently critical biomarkers to predict treatment response. The fusion protein from *ROS1* rearrangement is an emerging target.

In 2007, it was first reported that an inversion on chromosome 2p resulted in the creation of an *EML4*-*ALK* fusion gene in lung cancer [7]. Multiple *EML4-ALK* variants, represented by different *EML4* breakpoints, have been identified, as well as other fusion partners for *ALK*, such as *KIF5B* and *TFG* [8]–[10]. *ALK* rearrangements result in oncogenic fusions which lead to constitutive activity of the *ALK* tyrosine kinase with subsequent effects on proliferation, migration and survival [11]. Lung cancers harboring *ALK* rearrangements represent a unique subpopulation of lung cancer patients. The frequency of the *EML4*-*ALK* rearrangement ranges from 2% to 7% in unselected NSCLC patients [3], [12]. The frequency is higher in NSCLC patients with adenocarcinoma histology, non or light cigarette smoking history, and younger age, regardless of ethnicity [3], [12], [13]. However, these clinical characteristics are not shared by all carriers and molecular characterization is necessary to determine treatment eligibility [3], [14], [15].

*ALK* rearrangements are pharmacologically targetable with the small molecule tyrosine kinase inhibitor (TKI) crizotinib. In 2011, the FDA granted accelerated approval of crizotinib in response to the manifested clinical benefit.

Routine molecular diagnostics need to include evaluations for both *EGFR* mutations and *ALK* rearrangements [13], [15], [16]. It is expected that testing for *ROS1* rearrangements will be included soon. *ROS1* is another receptor tyrosine kinase that forms fusions in NSCLC and has shown responsiveness to crizotinib [17]. Diagnostic testing laboratories have been expected to rapidly introduce and perform molecular testing for NSCLC. For successful patient treatment, it is of great importance that molecular test results are accurate, highly reliable, and presented in a timely fashion. In 2012, the European Society of Pathology (ESP) proposed an external quality assessment (EQA) scheme to promote high quality biomarker testing in NSCLC for *EGFR* mutation analysis and *ALK* rearrangement detection. From 2014 on, *ROS1* testing is also included. The scheme aims to assess and improve the current status of molecular testing in NSCLC, to provide education and remedial measures, to permit inter-laboratory comparison and to allow validation of test methods by distributing validated material harboring well-defined aberrations. For *EGFR*, EQA results have been reported [18]. This article summarizes the results of the two *ALK* testing pilot rounds of the ESP Lung EQA scheme, organized in 2012–2013 with the purpose to reflect the current status of *ALK* rearrangement testing practices and to issue recommendations for the improvement of testing quality.

## Materials and Methods

A pilot EQA scheme consisting of two rounds was set up. Tissue microarray (TMA) slides that consisted of NSCLC cell-lines and resection specimens were distributed. Three expert laboratories (University of Groningen, the Netherlands, UK NEQAS ICC & ISH, United Kingdom and VU University Medical Center, Amsterdam) provided material for this EQA program. All patient samples were leftover tissues that were obtained as part of routine care and testing from the three laboratories mentioned above and then handed over to the researchers anonymously. These laboratories signed a statement that the patient material was obtained according to the national legal requirements for the use of patient samples. Informed consent is not a mandatory prerequisite for the use of patient derived material, since samples for test validation are exempt from research regulations requiring informed consent. The treating physician was responsible to obtain informed consent from the patients to use their tissues and data for research purposes and this consent is kept in the patient’s medical file. The authors had no contact with the patients or received any patient identifying information. The samples were to be analyzed for the presence of *ALK* rearrangements using IHC or FISH. In addition to the TMA slides, participation in *ALK* FISH included the interpretation of four digital FISH images which were accessible online. The *ALK* FISH digital cases were provided in close collaboration with UK NEQAS ICC & ISH.

In both rounds, mock clinical information was provided for several cases, for which the delivery of a full written report was requested. Report content was assessed in agreement with established standards/guidelines on reporting [19]–[21].

A central database was used for the submission of the results, accessible through the ESP Lung EQA Scheme website. Through their personal account, participants could access their results, scheme documentation and assessor feedback. Upon registration, each laboratory was assigned a unique EQA identity number to guarantee anonymity. A team of medical and technical experts supported the validation of the samples and the evaluation of the scheme results. Results were discussed during an assessment meeting to obtain final consensus scores. Participants received individual feedback and a general report with aggregated scheme results.

The set-up of both rounds slightly differed and the scheme was a pilot for the development and standardization of homogeneous testing material. Eight samples (four resection specimens and four cell lines) and twelve samples (six resection specimens and six cell lines) were prepared and send to the participants for respectively the first and second round. Different cell lines either with or without *ALK* break were routinely fixed with neutral-buffered formalin, mixed with agar and embedded in paraffin (reflecting routine pathology tissue block) were included. Results from samples for which less than 75% of the participants were able to obtain a result were not taken into account to assess performance [22]. Consequently, for *ALK* FISH, 3/8 and 7/12 samples were regarded as educational samples for respectively the first and second round. For the assessment, the accepted cases were two resection specimens and three cell lines for the first round of *ALK* FISH, and for the second round, five resection specimens were included. For *ALK* IHC, all samples were approved in both rounds.

For *ALK* FISH, it was requested for each case to report the number of neoplastic cell nuclei without hybridization signals, the number of neoplastic nuclei with fused signal, with split signal and with a single red signal. An algorithm automatically generated the number of neoplastic nuclei with FISH signal, the number of neoplastic nuclei with split or single red, and the fraction of FISH positive and negative nuclei. Participants were asked to then determine the outcome of the *ALK* FISH test (positive/negative). Samples for which a laboratory did not obtain results due to sample quality or technical failures were not assessed. For *ALK* FISH TMA and *ALK* FISH Digital, error rates were calculated based on the samples for which a minimum of 50 nuclei were enumerated, in order to exclude uninformative cases. This paper does not emphasize the marking criteria and assigned scores, as the scoring criteria slightly differed for the pilot rounds, but the study aims to reflect the current status of *ALK* rearrangement testing practices in molecular pathology laboratories.

For *ALK* IHC, it was requested to use the H-score procedure as described by Ruschoff et al. [23]. This modified H-score procedure is educational as it gives a better understanding of the *ALK* IHC sensitivity and reliability [14]. In the first round, a cut-off IHC score of 32 was determined by the mean score plus standard deviation from the laboratories on the IHC negative specimens (except outliers with H-score >100). The same threshold for positivity/negativity was applied in the second round.

For the statistical analysis, scheme error rates from both rounds for FISH digital and FISH TMA were compared using the Mann-Whitney U test. Scheme error rates for IHC were compared using an unpaired t-test. The level of significance was set at α = 0,05.

## Results

In total, 173 different laboratories (primarily from European Union countries) participated in the pilot rounds. In the first round, 29 laboratories submitted results for *ALK* IHC, 55 for *ALK* FISH TMA, and 67 laboratories performed the interpretation of the digital *ALK* FISH images. In the second round, 58 laboratories submitted results for *ALK* IHC, 104 for *ALK* FISH TMA, and 106 for the *ALK* FISH digital cases. For the data-analysis, missing values were ignored and only valid answers to questions were included, which explains why sample sizes slightly differ. Laboratory characteristics are listed in Table 1. The total number of laboratories that provided information was used as the denominator to calculate percentages. Because it was sometimes possible to indicate more than one answer, percentages may not add up to 100%.

**Table 1:** Laboratory characteristics.

Laboratory characteristics		Round 1		Round 2	
		N	% (n = 67)	N	% (n = 149)
Laboratory settings	Community Hospital	30	48%	80	54%
	University Hospital	24	36%	30	20%
	University	6	9%	17	11%
	Private	3	4%	16	11%
	Industry	2	3%	3	2%
	Private Hospital	0	0%	2	1%
	Other	2	3%	1	1%
Clinical tests offeredon lung biopsy samplesin routine diagnostics	*ALK* rearrangementdetection	56	84%	110	74%
	*EGFR* mutationdetection	/	/	128	86%
	*KRAS* mutationdetection	/	/	112	75%
Analysis performed underthe authority of thedepartment of pathology	Yes	61	91%	116	78%
	No	6	9%	33	22%
Tests most frequentlyordered on lung biopsies	*EGFR*	/	/	132	89%
	*ALK*	/	/	49	33%
	*KRAS*	/	/	44	30%
	None of the above	/	/	7	5%

The majority of the participants were set in a community hospital or university hospital environment. The analysis was mostly performed under the authority of the department of pathology. Regarding the interpretation of *ALK* FISH, a pathologist was most frequently involved (23% and 27% of laboratories in the first and second round, respectively), in some cases assisted by a scientist (18% and 27%) or a technician (20% and 13%). A scientist alone performed the FISH reading in 15% of the laboratories in both the first and second round. The final reading conclusion was the responsibility of a pathologist alone in more than half of the laboratories (52% and 59% in the first and second round). A pathologist in cooperation with a scientist was responsible in 13% and 14% of the laboratories. A scientist alone was responsible for the final reading conclusion in 16% and 12% of the laboratories in the first and second round.

### *ALK* FISH digital results

Results for both rounds of the *ALK* FISH digital subscheme are summarized in Table 2. There were no clear differences in the error rates depending on the number of nuclei enumerated. Enumeration practices were evaluated on sample level and on laboratory level. In both rounds, the bulk of the participants enumerated 50–100 nuclei for each case. At laboratory level, in the first round, 34/67 laboratories (51%) counted ≥50 cells for each sample. In the second round, an increase was observed to 77/106 (73%). Table 3 illustrates the performance of the labs that participated in both rounds for each subscheme. Improvement in enumeration practices was defined as enumeration of ≥50 cells in a larger number of samples in the second round compared to the first round.

**Table 2:** Results for the interpretation of digital *ALK* FISH images.

Round	SampleID	*ALK*rearrangementstatus	Number oflabs thatcounted<50 nuclei	Number oflabs thatcounted50–100 nuclei	Numberof labs thatcounted>100 nuclei	Number ofFP/FN fromlabs thatcounted<50 nuclei	Number ofFP/FN fromlabs that counted50–100 nuclei	Number ofFP/FN fromlabs that counted>100 nuclei
Round 1	LUNG12.109	negative	12 (18%)	38 (58%)	16 (24%)	1 (8%)	0 (0%)	1 (6%)
	LUNG12.110	negative	21 (32%)	43 (65%)	2 (3%)	0 (0%)	0 (0%)	0 (0%)
	LUNG12.111	positive	15 (23%)	45 (70%)	4 (6%)	0 (0%)	0 (0%)	0 (0%)
	LUNG12.112	positive	13 (22%)	37 (62%)	10 (17%)	3 (23%)	6 (16%)	0 (0%)
	Average		15,3 (23,8%)	40,8 (63,8%)	8,0 (12,5%)	1,0 (7,75%)	1,5 (4,0%)	0,3 (1,5%)
Round 2	LUNG12.213	negative	19 (18%)	74 (70%)	11 (10%)	0 (0%)	0 (0%)	0 (0%)
	LUNG12.214	positive	11 (11%)	69 (65%	24 (23%)	0 (0%)	0 (0%)	0 (0%)
	LUNG12.215	negative	10 (10%)	66 (62%)	27 (25%)	0 (0%)	1 (2%)	2 (7%)
	LUNG12.216	positive	9 (9%)	68 (64%)	27 (25%)	0 (0%)	1 (1%)	0 (0%)
	Average		12,3 (12,0%)	69,3 (65,3%)	22,3 (20,75%)	0 (0%)	0,5 (0,8%)	0,5 (1,8%)

FP, false positives; FN, false negatives.

**Table 3:** Performance of laboratories that participated in both rounds.

Subscheme	Nr of labs withimprovedenumerationperformance	Nr of labswith equalenumerationperformance	Nr of labswith worseenumerationperformance	Nr of labswith adecrease innr of errors	Nr of labswith equalnr of errors	Nr of labswith highernr of errors
*ALK* FISH Digital	14/39 (36%)	22/39 (56%)	3/39 (8%)	1/37 (3%)	36/37 (97%)	0/37 (0%)
*ALK* FISH TMA	13/35 (37%)	17/35 (49%)	5/35 (14%)	7/29 (24%)	20/29 (69%)	2/29 (7%)
*ALK* IHC	/	/	/	3/14 (21%)	9/14 (64%)	2/14 (14%)

A decrease was observed in the error rates between both rounds (error rates were calculated taking only the samples for which ≥50 nuclei were enumerated into account). In the first round, 7 out of 195 scored samples were incorrectly assigned (3,6%), while in the second round, 4 errors out of 366 scored samples (1,1%) occurred. The comparison of the number of errors made for laboratories that participated in both rounds and that counted ≥50 nuclei for each case can be found in Table 3.

### *ALK* FISH TMA results

Table 4 summarizes the *ALK* FISH TMA results for both rounds. In both rounds, the majority of the participants enumerated 50–100 nuclei for each case. Again, there were only small differences in the error rates depending on the number of nuclei enumerated. In the first round, 30/55 laboratories (55%) counted ≥50 cells for each sample; in the second round there was an increase to 81/104 (78%).

**Table 4:** *ALK* FISH TMA results.

Round	Sample ID	*ALK*rearrangementstatus	Number oflabs thatcounted<50 nuclei	Number oflabs thatcounted50–100 nuclei	Numberof labs thatcounted>100	Number ofFP/FN fromlabs thatcounted<50 nuclei	Number ofFP/FN fromlabs thatcounted50–100 nuclei	Number ofFP/FN fromlabs thatcounted>100 nuclei
Round 1	LUNG12.101	positive	11(22%)	37 (74%)	2 (4%)	2 (18%)	4 (11%)	0 (0%)
	LUNG12.105	positive	11 (26%)	26 (62%)	5 (12%)	2 (18%)	6 (23%)	1 (20%)
	LUNG12.106	negative	10 (19%)	34 (64%)	9 (17%)	1 (10%)	1 (3%)	0 (0%)
	LUNG12.107	positive	11 (21%)	34 (64%)	8 (15%)	0 (0%)	2 (6%)	0 (0%)
	LUNG12.108	negative	10 (20%)	34 (68%)	6 (12%)	0 (0%)	0 (0%)	0 (0%)
	Average		10,6 (21,6%)	33,0 (66,4%)	6,0 (12,0%)	1 (9,2%)	2,6 (8,6%)	0,2 (4,0%)
Round 2	LUNG12.202	positive	10 (11%)	46 (51%)	34 (38%)	1 (10%)	14 (30%)	1 (3%)
	LUNG12.204	negative	8 (9%)	48 (52%)	37 (40%)	0 (0%)	1 (2%)	2 (5%)
	LUNG12.206	negative	7 (8%)	56 (60%)	30 (32%)	0 (0%)	0 (0%)	1 (3%)
	LUNG12.208	positive	6 (6%)	55 (57%)	36 (37%)	0 (0%)	1 (2%)	1 (3%)
	LUNG12.210	negative	6 (7%)	48 (53%)	36 (40%)	0 (0%)	0 (0%)	1 (3%)
	Average		7,4% (8,2%)	50,6 (54,6%)	34,6 (37,4%)	0,2 (2,0%)	3,2 (6,8%)	1,2 (3,4%)

FP, false positives; FN, false negatives.

For the TMA cases there was also a decrease in the error rates between the two rounds. In the first round, 14 out of 193 scored samples were incorrectly assigned (7,3%), while in the second round, 22 errors out of 423 scored samples (5,2%) occurred. Comparison of the enumeration performance and number of errors made for laboratories participating in both rounds can be found in Table 3.

### *ALK* IHC results

Results for both *ALK* IHC rounds are provided in Table 5. In the first round, 30/230 scored cases (13,0%) were incorrectly called (false positive or false negative). In the second round, a decrease to 44/540 (8,2%) was observed. Table 3 illustrates the performance for the labs that participated in both IHC rounds.

**Table 5:** *ALK* IHC results.

Round	Sample ID	*ALK*rearrangementstatus	AverageIHC score	Standarddeviation IHCscore	Number of FP/FN
Round 1	LUNG 12.101	positive	154	53	2 (7%)
	LUNG 12.102	positive	113	73	6 (22%)
	LUNG 12.103	negative	15	51	2 (7%)
	LUNG 12.104	negative	24	49	5 (17%)
	LUNG 12.105	positive	118	61	3 (10%)
	LUNG 12.106	negative	25	53	6 (21%)
	LUNG 12.107	positive	154	62	2 (7%)
	LUNG 12.108	negative	13	32	4 (14%)
	Average	/	/	/	3,8 (13,1%)
Round 2	LUNG12.201	negative	18	52	5 (12%)
	LUNG12.202	negative	16	51	5 (11%)
	LUNG12.203	negative	5	22	2 (5%)
	LUNG12.204	negative	6	23	2 (4%)
	LUNG12.205	negative	18	47	7 (16%)
	LUNG12.206	negative	1	4	0 (0%)
	LUNG12.207	negative	9	34	4 (9%)
	LUNG12.208	positive	163	58	4 (9%)
	LUNG12.209	positive	137	69	7 (16%)
	LUNG12.210	negative	2	11	1 (2%)
	LUNG12.211	positive	143	68	6 (13%)
	LUNG12.212	negative	1	6	1 (2%)
	Average	/	/	/	3,7 (8,3%)

FP, false positives; FN, false negatives.

### Summary scheme error rates

The Mann-Whitney U test revealed no significant differences between both rounds for Digital FISH (U = 7, z = –0.308, p = 0.758) or FISH TMA (U = 9, z = –0.731, p = 0.465). For IHC, an unpaired t-test showed no significant difference between the first (M = 0.13, SD = 0.06) and second round (M = 0.08, SD = 0.05); t(18) = 1.845, p = 0.082. Although not statistically significant, comparing the error rates in both rounds suggests a learning effect (Table 6). The smallest error rates were observed for the digital cases, assessing only the post-analytical interpretation phase. In both rounds, the error rate for *ALK* FISH TMA was lower than the error rate for *ALK* IHC TMA.

**Table 6:** Scheme error rates per subscheme for both pilot rounds.

Subscheme	Error rate round 1*		Error rate round 2*	
Digital *ALK* FISH	3,6%	0,5% due to FP	1,1%	0,8% due to FP
		3,1% due to FN		0,3% due to FN
*ALK* FISH TMA	7,3%	0,5% due to FP	5,2%	4,0% due to FP
		6,7% due to FN		1,2% due to FN
*ALK* IHC	13,0%	7,4% due to FP	8,2%	5,0% due to FP
		5,6% due to FN		3,2% due to FN

*Error rate = (number of FP + number of FN)/total number of informative results FP, false positives; FN, false negatives.

### Methods used

The most frequently used method for FISH analysis was the Vysis *ALK* break apart FISH probe kit (Abbott Molecular, Illinois, USA), used by over 70% of the participants. For IHC, the most frequently used antibodies were clone 5A4 and clone D5F3 for the first and second round, respectively. An overview of the used methods and the error rate per method is provided in Tables 7 and 8. For the percentage of laboratories that used a certain method, the total number of laboratories that provided information was used as the denominator. Because it was possible to indicate more than one used method, percentages may not add up to 100%.

**Table 7:** *ALK* FISH methods used in the scheme and error rate per probe.

*ALK* FISHrearrangementdetection method	Round 1				Round 2			
	Percentage oflabs that usedthe probe (n = 60)	Number ofFP/FN obtainedwith the probe	Total numberof samplesanalysed withthe probe	Percentage ofsamples resultingin a FP/FN	Percentage oflabs that used theprobe (n = 103)	Number ofFP/FN obtainedwith the probe	Total number ofsamples analysedwith the probe	Percentage ofsamples resultingin a FP/FN
ALK FISH DNAProbe, Split Signal(Dako, Glostrup, Denmark)	12%	3	29	10,3%	11%	0	38	0,0%
ZytoVision(Bremerhaven, Germany),not further specified	/	/	/	/	2%	1	9	11,1%
Master Diagnostica(Granada, Spain)	/	/	/	/	1%	/	/	/
Repeat-FreePoseidon ALK(2p23) Break Probe(Kreatech Diagnostics, Amsterdam,the Netherlands)	2%	1	5	20,0%	3%	0	10	0,0%
Repeat-Free PoseidonALK/EML4 t(2;2)inv(2) Fusion Probe(Kreatech Diagnostics,Amsterdam, the Netherlands)	7%	0	16	0,0%	1%	2	4	50,0%
Vysis ALK break apartFISH probe kit(Abbott Molecular, Illinois, USA)	82%	11	157	7,0%	78%	16	311	5,1%
ZytoLight SPEC ALK DualColor Break Apart Probe(ZytoVision, Bremerhaven, Germany)	/	/	/	/	1%	0	4	0,0%
ZytoLight SPEC ALK/EML4TriChec Probe(ZytoVision, Bremerhaven, Germany)	7%	0	14	0,0%	7%	2	30	6,7%

FP, false positives; FN, false negatives.

**Table 8:** Antibodies used for *ALK* IHC in the scheme and error rate per antibody.

*ALK* IHC antibody	Round 1				Round 2			
	Percentage oflabs that usedthe antibody(n = 22)	Number ofFP/FN obtainedwith the antibody	Total numberof samplesanalysed withthe antibody	Percentage ofsamples resultingin a FP/FN	Percentage oflabs that used theantibody (n = 62)	Number ofFP/FN obtainedwith the antibody	Total numberof samplesanalysed withthe antibody	Percentage ofsamples resultingin a FP/FN
5A4	64%	6	104	5,8%	40%	5	185	2,7%
D5F3	14%	1	16	6,3%	44%	30	291	10,3%
ALK1	18%	9	32	28,1%	15%	7	35	20,0%
SP8	9%	8	16	50,0%	2%	1	11	9,1%
4C5B8	/	/	/	/	2%	1	6	16,7%

FP, false positives; FN, false negatives.

For *ALK* FISH, the Repeat-Free Poseidon ALK/EML4 t(2;2) inv(2) Fusion Probe (Kreatech Diagnostics, Amsterdam, the Netherlands) revealed a high error rate of 50% in the second round (Table 7). For IHC, the smallest error rates were observed for clones 5A4 and D5F3 (Table 8).

### Clinical result reporting

Evaluation of the written reports for the second round (n = 102) showed that a case-specific clinical interpretation was missing in 74% and 79% of the reports for an *ALK* positive and *ALK* negative case, respectively. Patient name and date of birth were correctly present in the majority of the reports, as well as a specification of the methods used (FISH kit information or IHC antibody). However, a specification of the aberrations tested and the threshold of the method were not mentioned in 46% and 47% of the FISH reports. The total number of neoplastic cells analyzed and the number of cells with split and/or single signal were missing in 23% of the FISH reports. For IHC, the threshold for positivity/negativity was not defined in 81% of the reports, and the staining intensity was missing in 39% of the reports.

## Discussion

Major advances have been made in the management of patients with NSCLC, with improved treatment response and survival following the introduction of molecular targeted TKI therapies focusing on *EGFR* mutations and *ALK* rearrangements. The increasing importance of morphology-based studies such as IHC or FISH has made the pathologist’s involvement a key element in precision medicine for NSCLC [2], [24].

In reply to the growing demands, laboratories have introduced molecular testing for NSCLC in routine diagnostics. Regular participation in quality assurance programs is crucial to ensure a high quality of testing service and to warrant patient safety [15], [18], [19].

Our results show that in the majority of the participating laboratories, *ALK* testing is performed under the authority of the pathology department. This is a necessity as FISH and IHC are both histological tests. Pathology review and assessment of section quality is essential considering the diversity and heterogeneity of tumor tissue [19], as false negatives may be due to poor fixation or insufficient neoplastic cell content [14], [18].

Three methods are generally used in routine diagnostics for *ALK* rearrangement detection: FISH, RT-PCR, and immunohistochemistry for aberrant expression of ALK protein [12], [14], [25]. Importantly, every assay should undergo validation in the laboratory before clinical interpretation and should be subject to regular internal and external quality controls [14], [19], [24]. The FDA approved test to determine *ALK* status is the Vysis LSI *ALK* dual color, break apart rearrangement probe (Abbott Molecular, Illinois, USA) [12], [14]. Although other IVD-CE labeled kits are available in Europe, this kit was by far the most frequently used method in both pilot rounds. *ALK* break apart (or split-signal) probes detect disruption of the *ALK* 2p23 locus but do not identify the partner fusion gene [3], [25]. Surprisingly, the *ALK/EML4* fusion probe is still occasionally used, although these probes miss the translocation of *ALK* with partners other than *EML4*. In the second round, the fusion probe revealed a high error rate of 50%. The cut-off values used during the clinical trials to prove the efficacy of crizotinib can be transferred from the Vysis probe to other break apart probes since the design (size + location) is highly similar [26]. The ZytoLight TriCheck (ZytoVision, Bremerhaven, Germany), used by approximately 7% of the participants in both rounds, can identify the presence of an *ALK* rearrangement and if the rearrangement partner is *EML4*. Today it is still under discussion whether it is important to know the fusion partner of *ALK* in relation to expected response to *ALK* TKIs [27], [28].

According to Abbott Molecular scoring criteria, a nucleus is considered positive if it contains at least one split signal or one isolated red signal. A first enumerator should count 50 nuclei. Cases with >50% and <10% positive nuclei are considered positive and negative respectively. If a sample shows between 10–50% positive nuclei, a second enumerator should also count 50 nuclei. If the average of the two readings contains at least 15% positive cells, the sample is considered positive. The kit specifies uninformative specimens as those in which fewer than 50 nuclei within the scribed area can be enumerated. In our evaluation these cases were therefore not included to calculate and compare the scheme error rates. It has been shown that the sensitivity and specificity of the kit increase as the number of tumor areas and number of nuclei scored increase [29], [30]. Our results showed that *ALK* rearrangement status was often determined on the evaluation of less than 50 nuclei by many participants. The percentage of false positive and false negative results upon enumeration of <50 nuclei did not reveal a clear difference compared to the percentage upon enumeration of ≥50 tumor nuclei. These findings correlate with the fact that the *ALK* rearrangement appears to be a homogenous event in the tumor population [26], [29], Enumeration of <50 nuclei is not advisable because this number is based on the minimal number that is statistically needed to be able to reliably define a sample without FISH break signals (<15% of nuclei) as a case without *ALK* rearrangement. In addition, the predictive value of phase III trial is based on this. Remarkably, some participants enumerated a large number of nuclei (e.g. >600 evaluated nuclei for case 12.215), which is not a requirement for daily practice. FISH interpretation should be performed in areas of the slide with clear signals, which are clearly distinct from the nuclear fluorescent ‘noise’ as well as from the background [15]. Importantly, selection of neoplastic nuclei is essential, and to this end sufficient morphological knowledge in FISH stained slides is obligatory, which stresses involvement of a pathologist.

It is not a surprise that the TMA FISH error rates were substantially higher than those of the digital FISH images. The FISH digital subscheme specifically assesses the interpretation of identical digital images whereas the TMA FISH error rate also incorporates variation in serial TMA sections, technical execution, and reading. Suboptimal *ALK* FISH procedure may lead to a low signal versus background ratio, increasing the chance for interpretation errors.

Although FISH is used as a standard test, it demonstrates considerable inter-observer variability. Therefore, experienced (>100 cases/year) and well-trained FISH reviewers/enumerators are necessary. If the clinical scientist is well trained and experienced in histo- and cytomorphology with specialized training in solid tumor FISH analysis, he/she can be responsible for the technical performance and molecular interpretation. A pathologist should at least be responsible for the selection of the right cells, the review of the interpretation and the authorization of the pathology report [14], [15]. Our data demonstrate that a pathologist was responsible for the final conclusion in the majority of the laboratories. Participating laboratories indicated that scientists and technicians were often involved in FISH enumeration. In this set-up it is important that the clinical scientist can consult a pathologist at any time in case of doubt concerning the location of the tumor cell area.

*ALK* IHC, if carefully clinically validated according to ISO 15189, may be considered as a screening method to select specimens for *ALK* FISH testing [15]. It is a cost-effective screening tool which correlated significantly with *ALK* FISH, using a number of antibodies including the 5A4 and D5F3 [25], [31]–[33]. However, discrepancies are reported also and need to be elucidated [34]. The 5A4 and D5F3 antibodies were the most frequently used clones in our study and revealed the smallest error rates, which is in accordance with literature [32], [33] and the findings of a recent NordiQC assessment [35]. Not surprisingly however, the error rates for IHC were greater than for FISH. Recently different validation projects for *ALK* IHC tests were done in collaboration with a lot of laboratories [36]. Moreover, on the website of NORDIQC (http://www.nordiqc.org/), advice on IHC staining protocols is given for several antibody clones.

Our study demonstrates improvement of *ALK* testing after only two EQA rounds. This suggests that laboratories constructively use the assessors’ feedback from the previous round to enhance their performance. Participation in EQA facilitates rapid exposure of errors and the timely implementation of corrective and preventive actions. However, other factors such as increased expertise and experience may play a part. It is expected that larger datasets, spanning a larger number of EQA participations will demonstrate a statistically significant improvement. On scheme level, the error rates for both *ALK* FISH and *ALK* IHC were lower in the second round and the *ALK* FISH digital scheme demonstrated an error rate of only 1,1%. Error rates for *ALK* FISH TMA and *ALK* IHC were still high (>5%), which stresses the need for continued education through EQA. Progress was also seen on individual laboratory level. For FISH analysis, improvements were observed both in the number of errors made and in enumeration practices.

Reporting of test results should take into account sample adequacy relative to the assay performance characteristics and limitations, and clinical reports should be readily interpretable by non-expert clinicians [19], [21]. Previous EQA schemes have exposed existing deficiencies in clinical reporting [18], [37]. Our results show that the content of reports for *ALK* rearrangement detection should be improved. Especially, a case-specific clinical interpretation, predicting the effect of the rearrangement status on therapy response, should be integrated in each report since a clear and concise assessment of the clinical implications of the result is crucial to fully inform treatment options.

Maintenance of quality assurance measures, including stringent internal quality controls and continued education by repeated EQA participations is essential to ensure high testing quality and rapid exposure of errors in order to warrant appropriate treatment choices. This article has demonstrated improvement in the performance of *ALK* FISH and *ALK* IHC in two consecutive EQA rounds. Several recommendations were made to improve the quality of *ALK* testing.

## References

[1] Jemal A, Bray F (2011) Center MM, Ferlay J, Ward E, et al (2011) Global cancer statistics. CA Cancer J Clin 61: 69–90. 21296855 10.3322/caac.20107

[2] Cagle PT, Allen TC, Olsen RJ (2013) Lung cancer biomarkers: present status and future developments. Arch Pathol Lab Med 137: 1191–1198. 23991729 10.5858/arpa.2013-0319-CR

[3] Kwak EL, Bang YJ, Camidge DR, Shaw AT, Solomon B, et al (2010) Anaplastic lymphoma kinase inhibition in non-small-cell lung cancer. N Engl J Med 363: 1693–1703. 20979469 10.1056/NEJMoa1006448 PMC3014291

[4] Stinchcombe TE, Socinski MA (2008) Gefitinib in advanced non-small cell lung cancer: does it deserve a second chance? Oncologist 13: 933–944. 18784157 10.1634/theoncologist.2008-0019

[5] Shepherd FA, Rodrigues Pereira J, Ciuleanu T, Tan EH, Hirsh V, et al (2005) Erlotinib in previously treated non-small-cell lung cancer. N Engl J Med 353: 123–132. 16014882 10.1056/NEJMoa050753

[6] Reck M, Heigener DF, Mok T, Soria JC, Rabe KF (2013) Management of non-small-cell lung cancer: recent developments. Lancet 382: 709–719. 23972814 10.1016/S0140-6736(13)61502-0

[7] Soda M, Choi YL, Enomoto M, Takada S, Yamashita Y, et al (2007) Identification of the transforming EML4-ALK fusion gene in non-small-cell lung cancer. Nature 448: 561–566. 17625570 10.1038/nature05945

[8] Choi YL, Takeuchi K, Soda M, Inamura K, Togashi Y, et al (2008) Identification of novel isoforms of the EML4-ALK transforming gene in non-small cell lung cancer. Cancer Res 68: 4971–4976. 18593892 10.1158/0008-5472.CAN-07-6158

[9] Takeuchi K, Choi YL, Togashi Y, Soda M, Hatano S, et al (2009) KIF5B-ALK, a novel fusion oncokinase identified by an immunohistochemistry-based diagnostic system for ALK-positive lung cancer. Clin Cancer Res 15: 3143–3149. 19383809 10.1158/1078-0432.CCR-08-3248

[10] Rikova K, Guo A, Zeng Q, Possemato A, Yu J, et al (2007) Global survey of phosphotyrosine signaling identifies oncogenic kinases in lung cancer. Cell 131: 1190–1203. 18083107 10.1016/j.cell.2007.11.025

[11] Dacic S (2013) Molecular genetic testing for lung adenocarcinomas: a practical approach to clinically relevant mutations and translocations. J Clin Pathol 66: 870–874. 23801495 10.1136/jclinpath-2012-201336

[12] Mino-Kenudson M, Mark EJ (2011) Reflex testing for epidermal growth factor receptor mutation and anaplastic lymphoma kinase fluorescence in situ hybridization in non-small cell lung cancer. Arch Pathol Lab Med 135: 655–664. 21526964 10.5858/2011-0029-RAI.1

[13] Sasaki T, Janne PA (2011) New strategies for treatment of ALK-rearranged non-small cell lung cancers. Clin Cancer Res 17: 7213–7218. 22010214 10.1158/1078-0432.CCR-11-1404 PMC3477548

[14] Thunnissen E, Bubendorf L, Dietel M, Elmberger G, Kerr K, et al (2012) EML4-ALK testing in non-small cell carcinomas of the lung: a review with recommendations. Virchows Arch 461: 245–257. 22825000 10.1007/s00428-012-1281-4 PMC3432214

[15] Lindeman NI, Cagle PT, Beasley MB, Chitale DA, Dacic S, et al (2013) Molecular testing guideline for selection of lung cancer patients for EGFR and ALK tyrosine kinase inhibitors: guideline from the College of American Pathologists, International Association for the Study of Lung Cancer, and Association for Molecular Pathology. J Mol Diagn 15: 415–453. 23562183 10.1016/j.jmoldx.2013.03.001

[16] Mescam-Mancini L, Lantuejoul S, Moro-Sibilot D, Rouquette I, Souquet PJ, et al (2014) On the relevance of a testing algorithm for the detection of ROS1-rearranged lung adenocarcinomas. Lung Cancer 83: 168–173. 24380695 10.1016/j.lungcan.2013.11.019

[17] Thunnissen E, van der Oord K, den Bakker M (2014) Prognostic and predictive biomarkers in lung cancer. A review. Virchows Arch 464: 347–358. 24420742 10.1007/s00428-014-1535-4

[18] Deans ZC, Bilbe N, O'Sullivan B, Lazarou LP, de Castro DG, et al (2013) Improvement in the quality of molecular analysis of EGFR in non-small-cell lung cancer detected by three rounds of external quality assessment. J Clin Pathol 66: 319–325. 23378269 10.1136/jclinpath-2012-201227

[19] van Krieken JH, Normanno N, Blackhall F, Boone E, Botti G, et al (2013) Guideline on the requirements of external quality assessment programs in molecular pathology. Virchows Arch 462: 27–37. 23250354 10.1007/s00428-012-1354-4

[20] International Organization for Standardization (2012) ISO 15189: 2012 Medical laboratories - Particular requirements for quality and competence. Geneva, ISO.

[21] Gulley ML, Braziel RM, Halling KC, Hsi ED, Kant JA, et al (2007) Clinical laboratory reports in molecular pathology. Arch Pathol Lab Med 131: 852–863. 17550311 10.5858/2007-131-852-CLRIMP

[22] Thunnissen E, Bovee JV, Bruinsma H, van den Brule AJ, Dinjens W, et al (2011) EGFR and KRAS quality assurance schemes in pathology: generating normative data for molecular predictive marker analysis in targeted therapy. J Clin Pathol 64: 884–892. 21947301 10.1136/jclinpath-2011-200163

[23] Ruschoff J, Dietel M, Baretton G, Arbogast S, Walch A, et al (2010) HER2 diagnostics in gastric cancer-guideline validation and development of standardized immunohistochemical testing. Virchows Arch 457: 299–307. 20665045 10.1007/s00428-010-0952-2 PMC2933810

[24] Garrido P, de Castro J, Concha A, Felip E, Isla D, et al (2012) Guidelines for biomarker testing in advanced non-small-cell lung cancer. A national consensus of the Spanish Society of Medical Oncology (SEOM) and the Spanish Society of Pathology (SEAP). Clin Transl Oncol 14: 338–349. 22551539 10.1007/s12094-012-0806-2

[25] Selinger CI, Rogers TM, Russell PA, O'Toole S, Yip P, et al (2013) Testing for ALK rearrangement in lung adenocarcinoma: a multicenter comparison of immunohistochemistry and fluorescent in situ hybridization. Mod Pathol26: 1545–1553. 10.1038/modpathol.2013.87 23743928

[26] Zwaenepoel K, Van Dongen A, Lambin S, Weyn C, Pauwels P (2014) Detection of ALK expression in NSCLC with ALK-gene rearrangements - comparison of multiple IHC methods. Histopathology. In press. 10.1111/his.12414 24621075

[27] Heuckmann JM, Balke-Want H, Malchers F, Peifer M, Sos ML, et al (2012) Differential protein stability and ALK inhibitor sensitivity of EML4-ALK fusion variants. Clin Cancer Res 18: 4682–4690. 22912387 10.1158/1078-0432.CCR-11-3260

[28] Crystal AS, Shaw AT (2012) Variants on a theme: a biomarker of crizotinib response in ALK-positive non-small cell lung cancer? Clin Cancer Res 18: 4479–4481. 22912388 10.1158/1078-0432.CCR-12-1952

[29] Camidge DR, Kono SA, Flacco A, Tan AC, Doebele RC, et al (2010) Optimizing the detection of lung cancer patients harboring anaplastic lymphoma kinase (ALK) gene rearrangements potentially suitable for ALK inhibitor treatment. Clin Cancer Res 16: 5581–5590. 21062932 10.1158/1078-0432.CCR-10-0851 PMC3395226

[30] Yoshida A, Tsuta K, Nitta H, Hatanaka Y, Asamura H, et al (2011) Bright-field dual-color chromogenic in situ hybridization for diagnosing echinoderm microtubule-associated protein-like 4-anaplastic lymphoma kinase-positive lung adenocarcinomas. J Thorac Oncol 6: 1677–1686. 21921848 10.1097/JTO.0b013e3182286d25

[31] Han XH, Zhang NN, Ma L, Lin DM, Hao XZ, et al (2013) Immunohistochemistry reliably detects ALK rearrangements in patients with advanced non-small-cell lung cancer. Virchows Arch 463: 583–591. 23955278 10.1007/s00428-013-1472-7

[32] Conklin CM, Craddock KJ, Have C, Laskin J, Couture C, et al (2013) Immunohistochemistry is a reliable screening tool for identification of ALK rearrangement in non-small-cell lung carcinoma and is antibody dependent. J Thorac Oncol 8: 45–51. 10.1097/JTO.0b013e318274a83e 23196275

[33] Mino-Kenudson M, Chirieac LR, Law K, Hornick JL, Lindeman N, et al (2010) A novel, highly sensitive antibody allows for the routine detection of ALK-rearranged lung adenocarcinomas by standard immunohistochemistry. Clin Cancer Res 16: 1561–1571. 20179225 10.1158/1078-0432.CCR-09-2845 PMC2831135

[34] Cabillic F, Gros A, Dugay F, Begueret H, Mesturoux L, et al (2014) Parallel FISH and Immunohistochemical Studies of ALK Status in 3244 Non-Small-Cell Lung Cancers Reveal Major Discordances. J Thorac Oncol 9: 295–306. 24518086 10.1097/JTO.0000000000000072

[35] Nordic Immunohistochemical Quality Control. Lung Anaplastic Lymphoma Kinase Assessment Run 39 2013. Available: http://www.nordiqc.org/Run-39-B16-H4/Assessment/Run39_ALK.pdf. Accessed 2014 Apr 1.

[36] Blackhall FH, Peters S, Bubendorf L, Dafni U, Hager H, et al (2014) Prevalence and Clinical Outcomes for Patients With ALK-Positive Resected Stage I to III Adenocarcinoma: Results From the European Thoracic Oncology Platform Lungscape Project. J Clin Oncol 32: 2780–2787. 25071109 10.1200/JCO.2013.54.5921

[37] Tembuyser L, Ligtenberg MJ, Normanno N, Delen S, van Krieken JH, et al (2014) Higher Quality of Molecular Testing, an Unfulfilled Priority: Results from External Quality Assessment for KRAS Mutation Testing in Colorectal Cancer. J Mol Diagn 16: 371–377. 24631467 10.1016/j.jmoldx.2014.01.003

